# Fine-tuned protein-lipid interactions in biological membranes: exploration and implications of the ORMDL-ceramide negative feedback loop in the endoplasmic reticulum

**DOI:** 10.3389/fcell.2024.1457209

**Published:** 2024-08-07

**Authors:** Tamir Dingjan, Anthony H. Futerman

**Affiliations:** Department of Biomolecular Sciences, Weizmann Institute of Science, Rehovot, Israel

**Keywords:** fine-tuning, sphingolipid, ceramide, endoplasmic reticulum, ORMDL

## Abstract

Biological membranes consist of a lipid bilayer in which integral membrane proteins are embedded. Based on the compositional complexity of the lipid species found in membranes, and on their specific and selective interactions with membrane proteins, we recently suggested that membrane bilayers can be best described as “finely-tuned molecular machines.” We now discuss one such set of lipid-protein interactions by describing a negative feedback mechanism operating in the *de novo* sphingolipid biosynthetic pathway, which occurs in the membrane of the endoplasmic reticulum, and describe the atomic interactions between the first enzyme in the pathway, namely serine palmitoyl transferase, and the product of the fourth enzyme in the pathway, ceramide. We explore how hydrogen-bonding and hydrophobic interactions formed between Asn13 and Phe63 in the serine palmitoyl transferase complex and ceramide can influence the ceramide content of the endoplasmic reticulum. This example of finely-tuned biochemical interactions raises intriguing mechanistic questions about how sphingolipids and their biosynthetic enzymes could have evolved, particularly in light of their metabolic co-dependence.

## 1 Introduction

Perhaps because of its philosophical and existential ramifications, the use of the term “fine-tuning” is not as widespread in biology as in physics and in cosmology, where it is used to describe the extraordinary precision of the physical and cosmological parameters that permit life to exist in our Universe (Barrow and Tipler, 1996). Recently, we attempted to reinforce the use of “fine-tuning” in the language of biology to describe atomic level interactions that are highly specific and highly regulated in living systems. With this in mind, we suggested that membrane lipid bilayers are “finely-tuned molecular machines,” based principally on the complexity and the specificity of the interactions that occur between membrane components (Dingjan and Futerman, 2021a). Thus, in addition to discussing the unanticipated compositional complexity of the lipid components of membrane bilayers, we also discussed how these lipids are able to interact with membrane proteins, not just by forming an “annular lipid boundary,” as was once thought (Gómez-Fernández and Goñi, 2022), but rather by specific atomic interactions that depend both upon the precise structure of the amphipathic lipid and on the precise sequence of amino acids within the protein structure (Dingjan and Futerman, 2021a).

In the current “Perspective,” we discuss one particular aspect of the modulation of protein function by lipids, so as to provide novel insight into the complex modes of interplay between these two classes of molecules. Our focus is on the regulation of sphingolipid (SL) synthesis in the endoplasmic reticulum (ER). SLs are one of the three major lipid classes in eukaryotic cell membranes (the others are sterols and glycerolipids) and are found across the tree of life, including in animals, plants, fungi and bacteria (Merrill, 2008; Futerman, 2021). SLs regulate a wide range of cellular processes including cell growth, immunity, inflammation, differentiation, tumor survival, and host-microbe interactions (Hannun and Obeid, 2018), and are also implicated in a number of human diseases (Dunn et al., 2019). Thus, their levels need to be carefully maintained within cells, and we now describe one of the cellular mechanisms by which SL levels are regulated, which involves a set of finely-tuned atomic interactions in the ER, where SLs are synthesized. This mechanism comprises a feedback loop involving an downstream metabolite that interacts with the first enzyme in the SL biosynthetic pathway. We suggest that this example of fine-tuning in biology should stimulate discussion about how such an extraordinarily precise system came into existence.

## 2 *De novo* ceramide synthesis in the ER

SLs are generated *de novo* by a set of stepwise enzymatic reactions beginning in the ER and continuing in the Golgi apparatus. SL synthesis depends on a wide variety of additional metabolites, known as components of the “anteome,” that is, those upstream metabolites and pathways without which SL synthesis could not occur (Santos et al., 2022; Biran et al., 2024b). We recently calculated that there may be as many as five pathways consisting of ∼28 enzymes and ∼40 metabolites that are absolutely required for SL biosynthesis in the ER (Santos et al., 2022), in addition to the transport mechanisms required to move SLs (ceramide) from the ER to the Golgi apparatus (Biran et al., 2024a).

**FIGURE 1:**
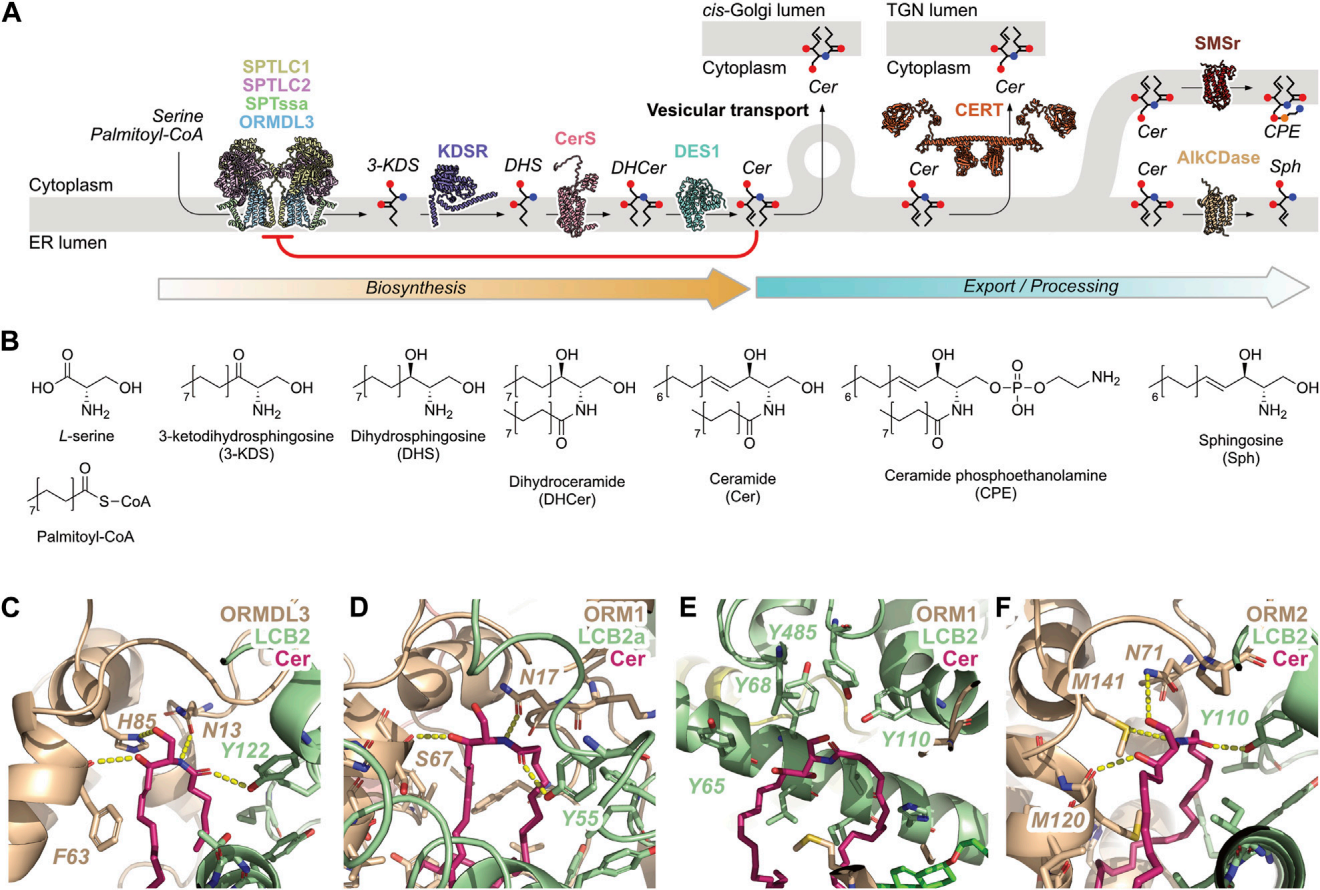
*De novo* Cer biosynthesis in the ER and its export/processing. **(A)** SLs are generated in the ER beginning with the generation of 3-ketodihydrosphingosine (3-KDS) by the SPT-ORMDL complex, followed by reduction to dihydrosphingosine (DHS) by 3-ketodihydrosphingosine reductase (KDSR), N-acylation by ceramide synthase (CerS), and desaturation by sphingolipid delta (4)-desaturase (DES1) to yield Cer. Once synthesised, Cer can be removed from the ER bilayer by vesicular transport to the *cis*-Golgi, or transported directly to the TGN by CERT. Cer can also be hydrolysed by alkaline ceramidase 1 (AlkCDase) to yield sphingosine and a fatty acid, or be further metabolized by SMSr to produce CPE. Cer exerts a negative feedback signal on SL biosynthesis by inhibition of the SPT-ORMDL complex, indicated by the red arrow. The concentration of Cer in the ER bilayer depends on the balance of reaction rates along the biosynthetic and export/processing routes. Protein models are shown in cartoon representation using coordinates from the AlphaFold Protein Structure Database. **(B)** Structures of SL species shown in panel **(A)**. **(C–F)** The ceramide-binding site at the LCB2/ORM interface in experimentally-determined eukaryotic SPT complexes. ORM proteins shown in taupe, LCB2 proteins shown in pistachio, Cer shown in fuchsia. Polar interactions are shown as yellow dashed lines. **(C)** *Homo sapiens* SPT-ORMDL3 complex (PDB ID: 7YIU), **(D)** *Arabidopsis* SPT-Orm1 complex (PDB ID: 7YJM), **(E)** *Saccharomyces cerevisiae* SPOTS-ORM1-Sac1 monomeric complex solved with Cer-44:0;4 (PDB ID: 8C81), **(F)** *Saccharomyces cerevisiae* SPT-Orm2 complex (PDB ID: 8IAJ).

The central player in SL metabolism is ceramide (Cer) which is found at the hub of the SL biosynthetic pathway (Hannun and Obeid, 2002; Zelnik et al., 2020) (Figure 1A). Cer is generated at the cytosolic leaflet of the ER (Hirschberg et al., 1993). If its levels become too high in the ER, ER stress ensues (Park and Park, 2020), characterized by the typical hallmarks of this process such as the unfolded protein response, the release of Ca^2+^ and apoptotic signalling (Senkal et al., 2010; Contreras et al., 2014; Liu et al., 2014; Qiu et al., 2020). Thus, the tight regulation of ER Cer content is crucial to maintain normal cell physiology (Breslow, 2013; Aguilera-Romero et al., 2014; Zelnik et al., 2020).

SL biosynthesis in the ER begins with the condensation of serine with an acyl-CoA, normally palmitoyl-CoA, by the serine palmitoyltransferase (SPT) complex, which is composed of a heterodimer of LCB1 and LCB2/3 (Hanada, 2003). Additional components of the SPT complex are not required for catalytic activity but enhance membrane localization and increase activity, specifically a small subunit, ssSPTa/b (Harmon et al., 2013), and an inhibitory subunit, ORMDL1/2/3 (Siow and Wattenberg, 2012) (Figure 1A). Alanine and glycine can also serve as substrates for SPT and their corresponding non-canonical SL products have been detected in yeast (Ren et al., 2018) and mammals (Zitomer et al., 2009; Steiner et al., 2016). Serine is the major substrate for mammalian SPT, reflected in the low levels of non-canonical SLs found in mammalian cells; increased usage of alanine and glycine by the SPT complex is caused by mutations associated with a sensory neuropathy HSAN1 (Gable et al., 2010; Harrison et al., 2018).

The SPT catalytic reaction commences with the binding of the amino acid which replaces an internal aldimine at the catalytic site-bound pyridoxal 5′-phosphate (PLP). Subsequent binding of an acyl-CoA facilitates the formation of a new bond between the serine α-carbon atom and the acyl-CoA thioester carbonyl carbon atom, releasing the coenzyme. Decarboxylation removes the C-terminus of serine as CO_2_, after which the product, 3-ketodihydrosphingosine (3KDS), is displaced from the active site by re-formation of the internal aldimine (Harrison et al., 2018) (Figures 1A, B). Following its generation, 3KDS is reduced by 3-ketodihydrosphingosine reductase (KDSR) in an NADPH-dependent manner to yield dihydrosphingosine (DHS), which is subsequently *N*-acylated by Cer synthase (CerS) which catalyzes the transfer of an acyl chain from acyl-CoA to the sphingoid long chain base to produce dihydroceramide (DHCer) (Kim et al., 2021) (Figures 1A, B). We have previously suggested that this mechanism may occur via a ternary complex in which both substrates are simultaneously bound to the enzyme (Zelnik et al., 2023). However, a recent preprint suggests that the CerS enzyme activity may instead proceed via a ping-pong mechanism, in which the acyl chain is first transferred from acyl-CoA to the enzyme, and subsequently to the sphingoid long chain base (Pascoa et al., 2023). Following *N*-acylation, DHCer is desaturated at the 4-5 carbon bond by sphingolipid delta(4)-desaturase (DES1) (Figures 1A, B). This reaction requires a reductant (either NADH or NADPH) and an electron donor in molecular oxygen (Michel et al., 1997). The enzyme is coupled to a series of electron-transport reactions to reduce the bound DHCer (Geeraert et al., 1997; Fabrias et al., 2012).

Further metabolism of Cer takes place in the ER via two additional reactions, namely synthesis of ceramide phosphoethanolamine (CPE) by SMSr, and hydrolysis of Cer by alkaline ceramidases (Figure 1A). In the former, SMSr catalyzes the transfer of phosphoethanolamine from phosphatidylethanolamine (PE) onto Cer to yield CPE and diacylglycerol (DAG) (Ternes et al., 2009; Vacaru et al., 2009; Tafesse et al., 2014) (Figures 1A, B). SMSr does not produce large amounts of CPE, suggesting that its role in the SL biosynthesis pathway is not the bulk production of CPE but rather to sense Cer levels in the ER lumenal leaflet, potentially via a conformational change during catalysis (Tafesse et al., 2014). Hydrolysis of Cer is catalyzed by members of the alkaline ceramidase family: AlkCDase-1 which localizes to the ER (Sun et al., 2008) and AlkCDase-3 which localizes to the ER and to the Golgi apparatus (Mao et al., 2001). AlkCDase-1 is expressed mainly in the skin and contributes to Cer homeostasis in the epidermis (Lin et al., 2017), while AlkCDase-3 regulates cell proliferation via the hydrolysis of Cer species containing long, unsaturated acyl chains (Hu et al., 2010). Whether the ER-localized AlkCDases can regulate Cer levels within the ER bilayer has yet to be demonstrated.

Finally, Cer can be removed from the ER bilayer via two transport mechanisms, vesicular transport to the *cis*-Golgi for the synthesis of glycosphingolipids (GSLs) (Biran et al., 2024a), and transport by the Cer transport protein CERT to the *trans*-Golgi network (TGN) for sphingomyelin (SM) synthesis (Hanada et al., 2003; Kumagai and Hanada, 2019) (Figure 1A). Thus, the SL *de novo* biosynthetic pathway in the ER consists of four enzymatic reactions that produce Cer (SPT through to DES1), two export routes that transport Cer to different regions of the Golgi apparatus, and two further enzymatic reactions that can metabolize Cer to possibly sense or regulate its levels (SMSr and AlkCDase).

## 3 Fine-tuning of Cer sensing by the SPT-ORMDL complex

We will now discuss a critical mechanism by which levels of Cer biosynthesis are regulated, presumably to avoid its toxic accumulation in the ER. This mechanism involves the sensing of Cer levels by ORMDL (Davis et al., 2018). ORMDL proteins (so named in mammals) are widespread across eukaryota, with Orm proteins in both yeast and *Arabidopsis* showing conserved function and sequence (Siow and Wattenberg, 2012). Human ORMDL proteins exist in three isoforms which share high sequence identity and are functionally redundant (Hjelmqvist et al., 2002; Siow and Wattenberg, 2012). The importance of Orm proteins in cell physiology is illustrated by the association of genetic variants of ORMDL3 to childhood asthma and to atherosclerosis (Moffatt et al., 2007; Ma et al., 2015), and the pathophysiology (dysmyelination and insulin resistance) observed in mouse knockout models (Clarke et al., 2019; Song et al., 2022). Human ORMDL proteins are expressed at higher levels than SPT (Siow et al., 2015), and their mechanism to sense Cer levels in the ER involves a direct response to the ER bilayer lipid composition (Davis et al., 2019).

Recent studies have reported the cryo-EM structures of the human Cer-bound SPT-ORMDL3 complex (Xie et al., 2023), the SPT-ORM1 complex of *Arabidopsis* (Liu et al., 2023), and the SPT-Orm-Tsc3-Sac1 complex (SPOTS complex) in *Saccharomyces cerevisiae* comprising LCB1, LCB2, Tsc3, Sac1, and Orm1/2 (Schäfer et al., 2023; Xie et al., 2024) [reviewed in (Maghram et al., 2023)] (Figures 1C–F). The current working model for how the SPT-ORMDL complex is inhibited by Cer involves Cer binding to the interface between LCB2 and the ORMDL/Orm protein. Upon binding to the human SPT-ORMDL complex, the highly-flexible N-terminal sequence of ORMDL is conformationally locked into an inhibitory conformation that prevents access by serine and palmitoyl-CoA to the catalytic site (Xie et al., 2023). In *Arabidopsis*, ceramide binding to the SPT-ORM1 complex induces the formation of a hybrid β sheet which stabilises the N-terminus of ORM1 in an inhibitory conformation (Liu et al., 2023). A similar mechanism was suggested for the yeast SPOTS-Orm2 complex, wherein Cer binding to the SPOTS-Orm2 interface, combined with dephosphorylation at the Orm2 N-terminus, stabilises the conformations of both N- and C-termini to block substrate access to the active site (Xie et al., 2024). The yeast SPOTS-Orm1 structure, however, features a different binding mode in which the bound Cer directly prevents substrate access to the SPT active site (Schäfer et al., 2023).

In all four of the structures, Cer is bound at the interface between LCB2 and the ORM-like protein. The binding site features multiple hydrophobic residues which interact with the Cer LCB and acyl chain hydrophobic regions, and polar residues which engage in hydrogen-bonding polar contacts with the first five carbon atoms of the LCB, i.e., the sphingoid motif (Dingjan and Futerman, 2021b). Of these, a highly-conserved Asn residue contributes significantly to Cer binding via a hydrogen bond to the 2-position amide (Asn13 in Figure 1C, Asn17 in Figure 1D) or the 1-position hydroxyl moiety (Asn71 in Figure 1F) (Xie et al., 2023; Xie et al., 2024). Additional hydrogen bonds are formed with the other polar atoms in the sphingoid motif such that the hydroxyl moiety at the 1-position interacts with His85 in the human SPT-ORMDL3 complex (Figure 1C) and the hydroxyl moiety at the 3-position with the backbone carbonyl oxygen of residue Phe63, Ser67, or Met120 (Figures 1C, D, F). A hydrogen bond is also donated to the Cer acyl chain carbonyl oxygen by Tyr122/55/110 from SPT LCB2 (Figures 1C, D, F). As noted above, the yeast SPOTS-ORM1 complex displays a different binding mode for Cer, in which the lipid contacts LCB2 residues Tyr485 and Tyr110 at the substrate access tunnel (Figure 1E).

Interestingly, the SPT-ORM complex is highly selective for Cer over other SLs. In the human SPT-ORMDL3 complex, SPT activity was inhibited by C6-Cer to ∼40% of wild-type (WT) activity, with weaker inhibition of complexes containing ORMDL1 or ORMDL2 (∼70% and ∼90% of WT activity). No inhibition was observed for Cer analogues and related SLs such as C6-DHCer, C6-1-deoxyCer, 3-KDS, DHS, sphingosine, and sphingosine 1-phosphate (S1P) (Xie et al., 2023). In *Arabidopsis*, C6-Cer inhibited the SPT-ORM1 complex to ∼40% of WT activity, C6-phytoCer inhibited more strongly (∼20% of WT activity), while C6-DHCer had no effect (Liu et al., 2023). In yeast, both C8-Cer and C8-phytoCer inhibited the SPOTS-Orm2 complex to a greater extent than C8-DHCer (Xie et al., 2024). The selectivity of the SPT-ORM complex for Cer over DHCer is consistent across multiple eukaryotes, highlighting the evolutionary conservation of the Cer-sensing mechanism.

Mutation of the hydrogen-bonding residues Asn13 and Phe63 to alanine in the human SPT-ORMDL3 complex increased SPT activity to approximately twice that of the WT complex, while mutation of Phe63 to arginine further increased activity to ∼4-fold that of WT, showing increased disruption of Cer binding (Xie et al., 2023). Mutation of His85 and Tyr122 to alanine, however, showed unchanged activity. The *Arabidopsis* SPT-ORM1 complex showed increased activity when Cer-binding residues were mutated: mutation of Asn17 to Ala increased activity to 2.5-fold WT levels as did mutation of residues Ser67 or Trp20 to arginine (Liu et al., 2023). Likewise the yeast SPOTS-Orm2 complex showed increased activity to between 2-fold and 4-fold WT levels with mutations to Asn71, Met120, and Met141 (Xie et al., 2024). Thus, there is a concrete mechanistic link between the affinity of the SPT-ORMDL complex for Cer and the inhibitory ability of Cer with respect to SL biosynthesis.

**FIGURE 2:**
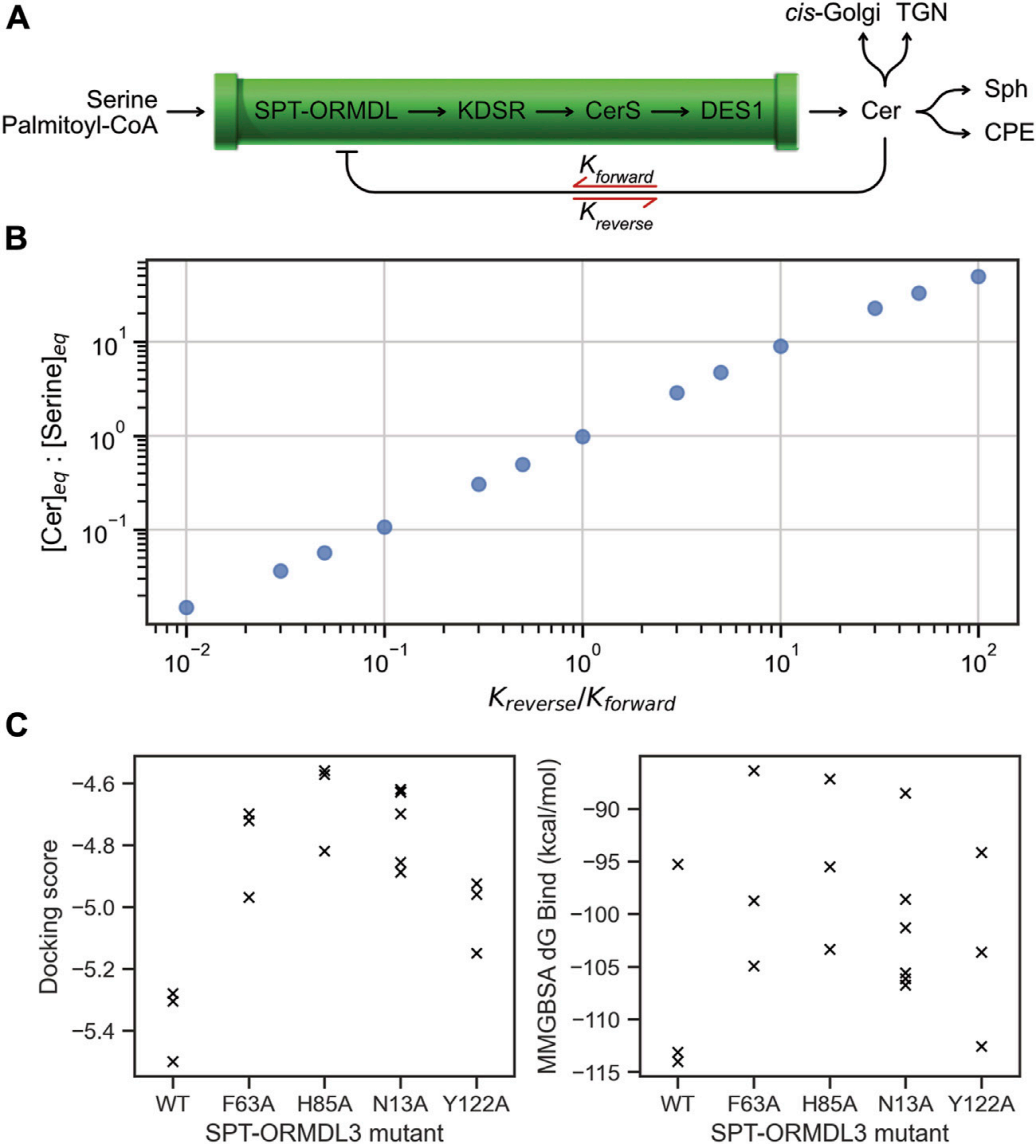
Computational modelling of the impact of SPT-ORMDL mutations on Cer levels. **(A)** A simplified representation of the SL biosynthetic and export/processing pathway. By considering the biosynthetic pathway as a single pipeline for the production of Cer in the ER, the inhibition of SL biosynthesis can be modelled as a mass-action process dependant on Cer sensing by the SPT-ORMDL complex. *K*_*forward*_ represents the equilibrium of Cer binding by the SPT-ORMDL complex, inhibiting synthesis, while *K*_*reverse*_ represents unbinding of Cer from the complex. **(B)** Mathematical modelling of the simplified SL biosynthetic pathway shows the changes in the equilibrium ratio of Cer:serine (vertical axis) caused by changes to the balance of the Cer inhibition reaction equilibria (horizontal axis). Values to the left of the horizontal axis correspond to a small K_reverse_:K_forward_, representing an inhibition equilibrium heavily favouring the inhibited SPT-ORMDL complex. This case describes sensitive inhibition of the SPT-ORMDL complex by Cer, resulting in reduced Cer levels at equilibrium compared to serine. By contrast, values to the right of the horizontal axis correspond to a large K_reverse_:K_forward_ ratio, representing an inhibition equilibrium that favors unbound Cer and an active SPT-ORMDL complex. This case models an insensitive inhibition scenario (for example, as is caused by mutations in the SPT-ORMDL Cer-binding site) and produces accumulation of Cer, reflected by higher levels at equilibrium compared to serine. Note that values are shown as ratios, and not in units of molar concentration. **(C)** Redocking of the experimentally-determined SPT-ORMDL3 Cer-bound complex (PDB ID: 7YIU) to WT and mutant SPT-ORMDL3 complexes shows the energetic contribution of individual binding site residues. The docking workflow predicted between 3-to-6 bound conformations for each SPT-ORMDL3 complex (horizontal axis), whose docking scores (left panel) and MM-GBSA free energy of binding values (right panel) are plotted (x). The vertical axes show the energetic favourability of Cer binding by the WT and mutant complexes (in both plots, greater negative values are more energetically favourable). Both the docking score and the free energy of binding show more positive values for the mutant complexes compared to the WT, indicating less favourable binding of Cer by the mutant complexes.

We now illustrate this mechanistic link with a simplified computational model of the SL biosynthetic pathway (Figure 2). While a detailed model of the cellular flux of SL species is not within the scope of this article, this type of model has been reported previously for human (Wronowska et al., 2015) and yeast SL metabolism (Alvarez-Vasquez et al., 2005). The model used herein was constructued using BioCRNpyler (Poole et al., 2022) and simulated using Bioscrape (Swaminathan et al., 2022). By representing the series of four biosynthetic enzymes that together produce Cer in the ER as a single pipeline (Figure 2A), we illustrate how the inhibitory ability of Cer at the SPT-ORMDL complex impacts the equilibrium concentration of Cer in the ER.

As detailed above, the inhibition of SPT-ORMDL by Cer involves the binding of Cer to the protein complex via multiple hydrogen-bonding and hydrophobic interactions. The binding reaction progresses in the “forward” direction with the formation of these atomic-scale interactions between the protein and lipid. Correspondingly, dissociation of Cer from the SPT-ORMDL complex is the “reverse” reaction direction. In the model presented here, the equilibrium constants describing the extent of Cer binding to SPT-ORMDL at equilibrium are described by K_forward_ and K_reverse_ (Figure 2A). The inhibitory activity of Cer is represented as a mass-action reaction converting the SPT-ORMDL complex from an active to an inactive state. The model assumes a constant inflow of serine and constant removal of Cer via a single saturatable export/processing mechanism that accounts for all the metabolic routes which decrease Cer levels in the ER. Simulating the effect of different values for the K_forward_ and K_reverse_ equilibrium constants shows variation in the equilibrium ratio of Cer to serine in the ER (Figure 2B). Where the reverse reaction dominates, the Cer binding equilibrium favors free Cer in the ER over Cer bound to the SPT-ORMDL complex. As a result, the SPT-ORMDL complex is not as strongly inhibited, and the equilibrium ratio of Cer to serine is increased. This scenario corresponds to the multiple mutations tested in eukaryotic SPT-ORMDL/Orm complexes detailed above; in the computational model, an increased Cer concentration of 2-fold compared to WT (such as was observed for the human SPT-ORMDL3 complex) corresponds to a 2-fold increase in the value of K_reverse_ compared to K_forward_.

While this model is a highly simplified rendering of the SL biosynthetic pathway, it serves to illustrate a simple point: the equilibrium concentration of Cer in the ER is determined by the balance of parameters describing Cer binding to the SPT-ORMDL complex, which in turn relies on specific atomic-scale binding interactions formed in the protein-lipid complex. To quantify the contributions of these interactions, we evaluated the effect of mutating specific amino acids which form hydrogen-bonds in the human SPT-ORMDL3 complex using molecular docking (Figure 2C). By re-docking the solved Cer molecule into the experimentally determined structure bearing a single mutation in the binding site with tight constraints (0.1 Å to the experimentally-solved bound Cer conformation), we evaluated the energetic contribution of single residues to the binding affinity for Cer at SPT-ORMDL3 using the Glide docking score (Figure 2C, left), and the predicted binding free energy as calculated by a MM-GBSA approach (Figure 2C, right) (Friesner et al., 2004). Each of the tested point mutations reduced the docking score and the predicted binding free energy relative to WT. The F63A and N13A mutations were both shown experimentally to decrease Cer affinity for the SPT-ORMDL3 complex resulting in 2-fold higher SPT activity; these mutations are each predicted to decrease Cer binding free energy by approximately 8 kcal/mol (Figure 2C, right). In the case of the Y122A mutation, which did not significantly alter SPT activity, the most favourable docked pose gave a predicted binding free energy similar to WT (Figure 2C, right), while the docking score was the closest mutant to the WT (Figure 2C, left). The H85A mutation, which was likewise shown experimentally to have negligible effect on SPT activity, was predicted to have a large impact on Cer binding free energy (Figure 2C, right). This aberration may be due to the use of tight constraints during docking to force reproduction of the solved bound conformation; conformational rearrangement of the H85A mutant binding site may allow the protein to accommodate Cer in a manner comparable to the WT SPT-ORMDL3 complex. In summary, the fine-tuning of Cer sensing by SPT-ORMDL3 is highlighted by the observation that a 2-fold increase in enzymatic activity upon mutation is caused by a shift in the equilibrium of the Cer binding reaction, which is effected by the removal of a single hydrogen bonding interaction with Asn13, contributing approximately 8 kcal/mol to the free energy of binding.

## 4 Discussion

In this Perspective, we discuss the fine-tuning of the molecular structure of the SPT-ORMDL:Cer negative feedback loop that determines the flux of Cer through the biosynthetic pathway. As has been previously noted (Maghram et al., 2023), Cer concentration within the ER bilayer is maintained within a tolerance defined by two bounds: the maximum concentration the ER can tolerate before induction of ER stress, and the minimum concentration required for the adequate production of downstream SL species (specifically SM and GSLs). We show here that this tolerance window rests on a fine-tuned sensing of Cer by the SPT-ORMDL complex, mediated by atomic-scale binding interactions that define the inhibitory ability of Cer. Such fine-tuned interactions, facilitating the binding of a product of a downstream enzyme to the first enzyme in the pathway, pose significant challenges for explaining how such a pathway could have emerged, and evokes our previous discussion of what came first in evolutionary history, the “Sphinx or the egg” (Biran et al., 2024b).

To reiterate, Cer is produced by the enzymatic activity of the CerS, and the level of Cer generated is subsequently sensed by the SPT-ORMDL complex, resulting in a negative feedback loop that inhibits *de novo* Cer biosynthesis. As the name implies, this loop only exists when both Cer synthesis and sensing occur simultaneously, i.e., when ORMDL and CerS are both expressed in a single cell. This interdependence suggests that ORMDL should have co-emerged along with the CerS. Synthesising Cer in a eukaryotic cell without a negative feedback mechanism (i.e., expressing CerS without ORMDL) risks ER integrity, since accumulation of Cer causes ER stress. Likewise, no specific selective pressure has been proposed to be able to drive the emergence of a negative feedback mechanism able to sense Cer in the absence of Cer synthesis (i.e., expressing ORMDL without CerS). The general evolution of cellular homeostatic systems has been suggested to be driven by the reduction of physiological noise in information transfer (Woods and Wilson, 2013), or by thermodynamic constraints fundamental to cellular energy balance (Wilson and Matschinsky, 2021); however the conditions underlying the origin of many specific proteins involved in individual cellular homeostatic systems are entirely unknown. We suggest that in the case of ORMDL proteins, their regulatory role in SL synthesis likely required their co-emergence with other members of the SL biosynthetic enzyme cohort.

The selective sensitivity of human ORMDL proteins towards D-erythro Cer is also notable, and is more strictly selective than DES1 and SM synthase (both of which can accommodate L-threo stereoisomers of their substrates) (Venkataraman and Futerman, 2001). Likewise, the selectivity of ORMDL for Cer over DHCer is a curiosity, since the highly-similar DHCer is at least theoretically able to form similar intermolecular interactions as Cer within the SPT-ORMDL complex. Is this selectivity for Cer widespread across eukaryota, or a function of a later evolutionary development? We posit that the central role of Cer at the crossroads of SL metabolism confers a markedly stronger fitness advantage to negative feedback circuits that respond to Cer alone. If this can be demonstrated, this bias towards selective sensing of Cer would have striking implications for the original emergence of ORMDL, providing a strong selective pressure for the SPT-ORMDL complex to be highly selective for a single metabolite close to its inception.

Beyond the ER, the trafficking and transport pathways that move Cer to the Golgi apparatus are also critical in SL homeostasis (Olson et al., 2016). Flux through vesicular transport and CERT-mediated transport leads to two separate pools of Cer in the *cis*-Golgi and TGN. Each of these pools leads to very different metabolic destinations: Cer in the *cis*-Golgi undergoes successive glycosylation reactions to assemble a range of complex GSLs, while the TGN is the main site of SM synthesis (Biran et al., 2024a). While our simple metabolic model does not take into account differences in flux between these two Cer transport routes, a more detailed rendering of SL metabolism may help reveal the tolerance of each of these routes to Cer levels. Defects in CERT autoregulation underlie clinical conditions associated with genetic variants of CERT1 (Gehin et al., 2023); further experiments are needed to evaluate how the balance of Cer flux through these two routes may be regulated.

Perturbation of the finely-tuned ORMDL-Cer binding reaction has physiological and biomedical implications. Amyotrophic lateral sclerosis patients carrying mutations in the SPT LCB1 N-terminal transmembrane domain show increased serum SL levels (Mohassel et al., 2021). While not contributing residues to the Cer binding site, the LCB1 N-terminal transmembrane domain makes extensive contact with ORMDL3 within the ER bilayer. Mutations in this domain interfere with ORMDL3-mediated inhibition, reducing the inhibitory effect of Cer on the SPT complex and disrupting SL metabolism, possibly driving pathogenicity (Mohassel et al., 2021). The contribution of ORMDL3 expression to risk of childhood asthma (Moffatt et al., 2007) is less clear, with transgenic mice showing airway remodelling consistent with asthmatic disease (Miller et al., 2014) and no impact of ORMDL3 expression on asthma responses in allergen tests (Debeuf et al., 2019). Future work to elucidate the mechanistic connections between SL levels, SPT activity, and ORMDL protein expression is warranted to resolve the physiological roles of ORMDL proteins.

In conclusion, this Perspective has explored the relationship between mechanisms of Cer biosynthesis and sensing within the ER. By examining the dependence of SL levels on the sensitivity of human ORMDL proteins, we highlight the fine-tuning of SL homeostatic regulation. The simplified mathematical model constructed for SL flux in the ER demonstrates how decreased ORMDL sensitivity toward Cer can lead to accumulation of SL levels in the ER, though further detailed studies are necessary to elucidate the tolerances involved. We detail how mutations which decrease ORMDL sensitivity toward Cer through the ablation of key hydrogen-bonding and hydrophobic interactions act via energetic changes on the order of 8 kcal/mol to alter the fine-tuned Cer-binding reaction equilibrium. The findings highlight the importance of atomic-scale interactions for the maintenance of Cer concentrations within specific bounds to avoid toxic stress while ensuring the production of downstream sphingolipid species. The selectivity of the ORMDL negative feedback circuit for Cer suggests a high degree of co-emergence during the evolutionary development of the SL biosynthetic pathway. Further work to examine the degree of fine-tuning in the parameters governing the biosynthesis, transport and processing of Cer within the ER bilayer is warranted.

## Data Availability

The raw data supporting the conclusions of this article will be made available by the authors, without undue reservation.
